# Physician’s Knowledge and Attitudes on Antibiotic Prescribing and Resistance: A Cross-Sectional Study from Hail Region of Saudi Arabia

**DOI:** 10.3390/healthcare11111576

**Published:** 2023-05-27

**Authors:** Khaled Almansour, Jonaid Ahmad Malik, Ishfaq Rashid, Sakeel Ahmed, Mir Aroosa, Jehad M. Alenezi, Mohammed A. Almatrafi, Abdulmajeed A. Alshammari, Kashif Ullah Khan, Sirajudheen Anwar

**Affiliations:** 1 Department of Pharmaceutics, College of Pharmacy, University of Hail, Hail 55476, Saudi Arabia; 2 Department of Pharmacology and Toxicology, National Institute of Pharmaceutical Education and Research, Guwahati 781101, India; 3 Department of Biomedical Engineering, Indian Institute of Technology Ropar, Rupnagar 140001, India; 4 Department of Pharmacy Practice, M.M. College of Pharmacy, Maharishi Markandeshwar University, Mullana-Ambala 133207, India; 5 Department of Pharmacology and Toxicology, National Institute of Pharmaceutical Education and Research, Mohali 160062, India; 6 Department of Pharmacology, Jamia Hamdard University, New Delhi 110062, India; 7 Department of Clinical Pharmacy, College of Pharmacy, University of Hail, Hail 55476, Saudi Arabia; 8 Department of Pharmacology and Toxicology, College of Pharmacy, University of Hail, Hail 55476, Saudi Arabia

**Keywords:** antibiotic resistance, prescribing pattern, physician’s attitude, Hail region, Saudi Arabia

## Abstract

Background: Antibiotic (AB) resistance is caused partly by overuse, varies by region, and is influenced by prescriber perspectives. This study sought to determine physicians’ knowledge and attitudes toward AB prescribing, particularly in the Hail region of Saudi Arabia. Methods: An interdisciplinary team created and validated an electronic questionnaire via the test–retest method that measured reliability and consistency. The 19 questions covered the following subjects: demographic information (7), experience with AB resistance in daily work (3), AB prescribing behavior (2), communication with patients regarding AB resistance (3), and prescribing practices (4). The revised questionnaire was prepared and distributed to physicians in the Hail region via multiple electronic communication channels. Inferences were drawn based on descriptive statistics and multivariate regression analysis. Results: The questionnaire responses of 202 participants were eligible for analysis. A total of 70 (34.80%) participants were general practitioners, 78 (38.12%) were engaged in daily work that was only mildly related to AB resistance, and 25 (12.37%) performed work that was substantially related to AB resistance. A total of 88 (43.56%) physicians believed that prescribing behavior contributed to the emergence of AB resistance, whereas 68 (33.66%) did not. Regarding exposure, 51 (25.24%) physicians reported encountering instances of AB resistance monthly, whereas 104 (51.48%) reported seeing cases of AB resistance very rarely. In terms of prescribing practices, 99 (49.0%) physicians prescribed ABs to patients daily and 73 (36.13%) weekly. Regarding AB-resistance-related communication with patients, 73 (36.13%) physicians frequently discussed AB resistance with patients suffering from infections, whereas 13 (6.4%) never discussed it with patients. Conclusion: General practitioners in the Hail region exhibited comprehensive awareness of the elements that contribute to AB resistance but only rarely communicated about the issue with their patients, presuming the latter to be oblivious to the science behind AB resistance. Our findings suggest that the features underlying practitioners’ AB prescribing behavior could be a powerful strategy for lowering AB resistance.

## 1. Introduction

Antibiotics (ABs) are the most commonly prescribed drugs worldwide [1]. They have successfully extended the average life expectancy and quality of life by reducing the threat and transmission of infectious diseases [2]. However, ABs are frequently mishandled, which has resulted in a large number of resistant bacterial strains [3]. Recognition that excessive AB prescribing contributes to AB resistance has led to calls for reform, even though the optimum tactics for dealing with this problem remain unknown [4]. The effectiveness of ABs in preventing and treating illnesses is being compromised by rising bacterial resistance globally [5]. As a consequence, efforts must be made to improve physicians’ attitudes toward AB prescribing [6], which necessitates understanding physicians’ knowledge, attitudes, and behaviors regarding AB resistance. Indeed, it has been postulated that physicians’ knowledge, attitudes, and abilities are related to diminishing AB resistance and modifying AB prescribing behavior [7].

Antimicrobial resistance (AMR) has become a global public health issue, imposing a huge economic and clinical burden on European hospitals due to the estimated 25,000 related deaths per year [1]. In the United States of America alone, AMR caused 35,000 deaths in 2018, while the death rate associated with AMR in Thailand and India exceeded 38,000 and 58,000, respectively [2]. Bacterial resistance is linked to how patients use ABs [3]. ABs are easy to obtain in several countries, such as Sudan, Pakistan, Yemen, Saudi Arabia, the United Arab Emirates, and Brazil, where they are readily available to the general public without any regulation [4,5,6,7]. Currently, in Saudi Arabia, the prescribing of ABs is highly regulated (SFDA 2017).

AMR may result from AB misuse, including patient noncompliance with AB treatment regimens and self-medication practices, particularly when utilizing low-cost broad-spectrum ABs as a first-line therapy [4]. Prior studies that investigated physicians’ AB prescribing patterns revealed that many prescriptions were not based on scientific evidence, and most were doubtful [8]. When physicians are concerned about the spread of infection, the development of problems, or satisfying patients’ expectations, it has been observed that they may breach the standards of best clinical practices [9]. Such behaviors can play a role in the spread of AMR. Physicians’ uncertainty regarding etiology, the pressures associated with an intensive workflow, and patients’ desire for AB therapy represent other factors that can lead to an increase in the incidence of AMR [10]. The Saudi Laws Compendium prohibits pharmacists from dispensing ABs without a physician’s prescription. Yet, pharmacists have continued to dispense ABs, leading to a rise in AMR [11,12]. In 2018, the Saudi Ministry of Health declared that it would begin to enforce the executive regulations contained within the Health Practice Law, which restricts the dispensing of ABs without a prescription. Those who do not comply with the legislation will be penalized financially [7].

In Saudi Arabia, earlier AMR research concentrated on the general population’s knowledge and attitudes concerning self-medication with ABs and on physicians’ perspectives on AB prescribing [13,14,15,16]. However, no such studies have been conducted in the Hail region of Saudi Arabia. This study sought to investigate physicians’ attitudes toward AB prescribing as well as to identify the elements that foster such attitudes among physicians in Hail.

## 2. Methodology

### 2.1. Study Procedure

An electronic questionnaire written in English was designed following a thorough literature review of related topics targeting physicians in the Hail region. The questionnaire-based survey of physicians included general practitioners (GPs) and specialists in pediatrics, psychiatry, cardiology, ophthalmology, and obstetrics and gynecology. The questionnaire comprised 19 questions grouped into 5 parts: demographic information (7 questions), experience with AB resistance in daily work (3 questions), AB prescribing behaviors (2 questions), communication with patients regarding AB resistance (3 questions), and information guiding AB prescribing practices (4 questions). Most of the questions were designed to allow only one answer to be selected by respondents, although a few allowed for multiple answers. Scientists at the University of Hail reviewed the questionnaire to determine its content validity. Subsequently, a pilot test was performed with 10 experienced physicians to check the questionnaire’s viability and the clarity of the questions. The pilot test participants were asked to explain the topic of each question in their own words to boost the internal validity of the questionnaire. The physicians (*n* = 10) who took part in the pilot test were not included in the final analysis. They were also not affiliated with the physicians who developed the questionnaire. A list of the questions is provided in the supplementary materials. The physicians who participated in the study were recruited by visiting various clinics in the Hail region of Saudi Arabia.

### 2.2. Study Population

The sample population comprised physicians who willingly volunteered to participate in the research. The physicians included in the sample worked in a variety of government and private hospitals, primary healthcare centers and clinics. The sample size was calculated using the following formula:

$$n=\frac{z^{2}\times p\times q}{d^{2}}$$

where *n* is the required sample size, *p* is the prevalence, *z* is a constant (1.96), and *q* is (1-*p*). The minimum estimated sample size was determined with a 95% confidence interval (CI) ± 5 marginal error. According to the Ministry of Health [17], the number of physicians in the Hail region is 1479, meaning that the sample size for this study was calculated by the Survey System Calculator as 306 [18]. The questionnaire was sent to 306 potential participants and 202 responses were received, indicating a response rate of 66%.

### 2.3. Questionnaire Validation

The physicians who participated in the pilot test were not included in the final survey and related analysis. The internal reliability of the questionnaire was assessed via Cronbach’s alpha analysis [19]. This is a useful approach for characterizing the degree to which all the items in a particular category share the same idea or construct, and it is thus linked to the test items’ interrelatedness [20]. The allowable range of alpha values is 0.70–0.95. A limited number of questions, insufficient item interrelatedness, and heterogeneous constructs can all contribute to a low alpha value. If a lack of association between objects is the source of a low alpha value, specific elements should be modified or omitted. By contrast, if the alpha is too high, it could mean that some items are repetitive because they assess the same question differently. The maximum alpha value is suggested to be 0.90 [20]. According to our assessment of the questionnaire’s reliability, Cronbach’s alpha value was 0.73. The test-retest reliability was also calculated based on the data from 10 physicians, and the intraclass correlation coefficient (ICC) value was found to be 0.5 (Table 1).

### 2.4. Ethical Considerations

Ethical approval to conduct the study was obtained from the Institutional Review Board (IRB) of the Hail region’s General Directorate of Health Affairs (approval number H-08-L-074). All data were collected from clinicians and kept confidential. The survey was carried out in Saudi Arabia’s Hail region. This investigation involved no personal information.

### 2.5. Statistical Analysis

Descriptive statistics were mostly used to describe the gathered data on demographic factors and the responses to questions about attitudes toward AB prescribing. The data were summarized as frequencies (*n*) and percentages (%) for the categorical variables. The chi-squared test was used to look for differences between the categorical variables. A *p*-value of 0.05 was considered statistically significant. A logistic regression analysis was performed for each question to estimate the predictors of the responses. Moreover, sociodemographic factors and a variable for subjective involvement were used as predictors. Cronbach’s alpha test was employed to assess the questionnaire’s reliability.

## 3. Results

### 3.1. Participant Characteristics

Most participants (*n* = 124) were male, and the participants’ mean age was 40 years (range: 25–60 years). Physicians with all levels of experience were carefully chosen from the specialties of medicine, surgery, pediatrics, and obstetrics and gynecology. Self-administered questionnaires were completed electronically. In total, 202 physicians completed the demographic survey, comprising GPs and physicians specialized in internal medicine; obstetrics and gynecology; ear, nose, and throat (ENT); pulmonology; and pediatrics (34.8%, 9.5%, 11.9%, 5.0%, 6.0%, and 7.0%, respectively). The physicians’ sectors of employment were general or specialized government hospitals (*n* = 112, 56.3%), government primary care centers (*n* = 32, 16.1%), and private hospitals or clinics (*n* = 55, 27.6%) (Table 2).

Among the participating physicians (*n* = 202), 110 had been in practice for more than 10 years; 43, between 7 and 10 years; 32, between 4 and 6 years; 14, between 1 and 3 years; and 3, less than 1 year. The majority of the physicians (*n* = 50) visited clinics 301–500 times every 3 months (95% CI: 0.84–19.7; *p* = 0.001) (Table 3).

### 3.2. AB Resistance Experience in Daily Work

**Figure 1:**
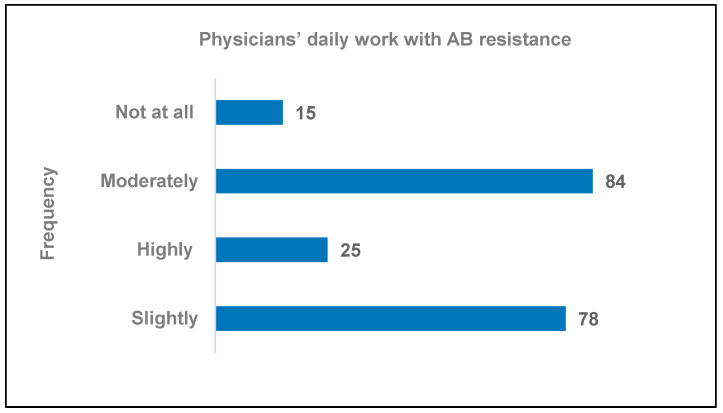
Frequency of encounters with AB resistance for physicians whose daily work is associated with AMR.

Among the 202 physicians who completed the questionnaire, 15 (7.32%) reported having no experience with AB resistance in their line of work (95% CI: 1.36–13.48; *p* < 0.05), 78 (386.12%) reported having only slight experience (95% CI: 10.3–66.92; *p* < 0.05), 84 (41.58%) reported having a moderate amount of experience (95% CI: 1.28–43), and 25 reported having a lot of experience (95% CI: 0.91–13.40; *p* < 0.05) (Figure 1).

### 3.3. Views on the Impact of AB Prescribing Behavior on the Development of AB Resistance

A total of 88 physicians (43.56%) believed that their AB prescribing behavior influenced the development of AB resistance among patients in the Hail region, whereas 68 (33.66%) did not. By contrast, 46 (22.77% of physicians) were unsure whether their prescribing behavior influenced the development of AB resistance. A total of 27 (13.3%) physicians faced AB resistance cases weekly, 104 (51.48%) rarely faced AB resistance cases, 51 (25.24) faced AB resistance cases monthly, and 19 (9.40%) never encountered the issue of AB resistance. Only one physician reported experiencing AB resistance on a daily basis.

### 3.4. AB Prescribing Behavior

Among the 202 physicians, 99 (49.0%) prescribed ABs to patients daily, 73 (36.13%) prescribed ABs weekly, and 12 (5.94%) prescribed ABs monthly. Only a small number of physicians (*n* = 18, 8.91%) rarely prescribed ABs to patients.

### 3.5. Communication with Patients Regarding AB Resistance

**Figure 2:**
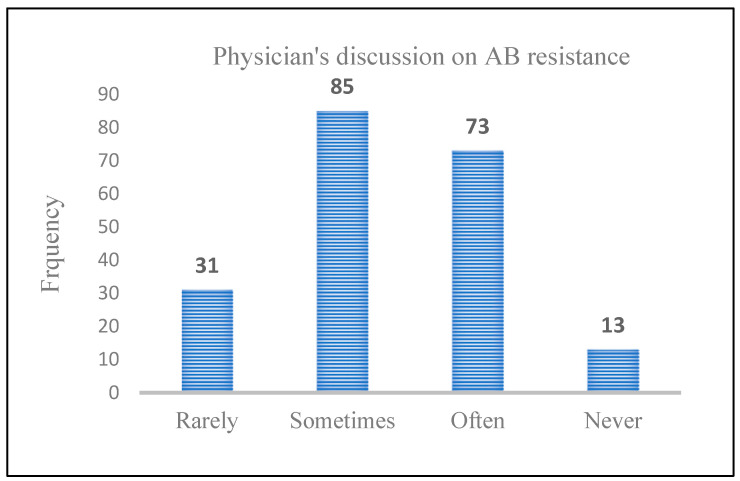
Frequency of physicians’ discussions of AB resistance with patients.

A small number of physicians (*n* = 13, 6.4%) never discussed AB resistance with infected patients, while 73 (36.13%) physicians were frequently involved in AB-resistance-related discussions with infected patients. In addition, 31 (15.34%) physicians rarely discussed AB resistance with patients suffering from infections, whereas 85 (42.0%) sometimes made an effort to discuss this issue (Figure 2).

### 3.6. Prescribing Practices

When questioned about the factors they thought would reduce the development of AB resistance, 17 (8.4%) physicians suggested AB prescription policies and protocols, 20 physicians posited the indications for ABs, while 10 (9.9%) physicians proposed the types of ABs used in hospitals, the ABs prescribed by GPs, and the use of ABs in animals and plants. Additionally, 148 (3.2%) physicians believed that patients’ knowledge and attitudes concerning AB use could reduce the development of AB resistance.

### 3.7. Delayed AB Prescribing Strategy and Practice Guidelines

This study also evaluated both physicians’ use of the delayed antibiotic prescribing strategy and their adherence to practice guidelines regarding AB therapy during their daily work. Among the 202 participating physicians, 98 (48.5%) physicians applied delayed AB prescribing on occasion, while 21 (10.3%) physicians rarely used any strategy for delayed AB prescribing. A total of 62 (30.6%) physicians often used a delayed AB prescribing strategy, whereas 15 (7.4%) physicians were unaware of any delayed AB prescribing strategies, and 6 (2.9%) physicians never used a delayed AB prescribing strategy.

In terms of their adherence to AB therapy practice guidelines, half of the physicians (*n* = 101, 50.0%) used them frequently, 80 (39.6%) physicians used them moderately, 10 (4.9%) physicians used them rarely, and 11 (5.4%) physicians never followed the guidelines in their daily work.

In this study, the use of a delayed prescribing strategy and adherence to practice guidelines were evaluated based on the physicians’ experience related to patients refilling their prescription; patients taking another AB within the first two weeks of the visit; ABs being collected before the recommended day; ABs being collected immediately; and inappropriate AB prescribing, which included needless AB prescribing, recommendation of an incorrect AB (inappropriate selection), administration of an incorrect dose, or ABs being prescribed for an incorrect length of time.

### 3.8. Reasons for Avoiding Discussing AB Resistance with Patients

This study revealed concerns regarding the discussion of AB resistance with patients. Among the 202 physicians, 49 (24.25%) physicians mentioned that discussing AB resistance might trigger patients’ worries, 29 (14.35%) physicians considered that patients would not understand any discussion of AB resistance, 61 (30.19%) physicians mentioned that they always discussed AB resistance with patients, and 62 (30.69%) physicians thought that insufficient time was a reason for avoiding discussing AB resistance with patients.

### 3.9. Reasons for ABs Being Prescribed without Clear Clinical Indications of Disease

This study also examined the causes of ABs being prescribed without any distinct therapeutic justification. More than half of the physicians (*n* = 104, 51.4%) stated that confirmatory diagnosis tools or methods were not available or were expensive, 16 (7.92%) physicians cited patients’ demand for ABs, 13 (6.43%) physicians suggested patients’ desire to get well quickly, 32 (15.84%) physicians mentioned patients’ noncompliance with other advice and treatments, 89 (44.05%) physicians suggested disease signs and symptoms being difficult to predict, and 9 (4.45%) physicians believed that difficulty communicating with patients (language barriers or cognitive impairments) explained the prescription of ABs without clear clinical indications of disease.

### 3.10. Views on Evidence-Based Therapy Guidelines for AB Prescription

The physicians were asked about the use of evidence-based prescription guidelines for ABs. A total of 186 (92.07%) physicians reported being in favor of such guidelines, 11 (5.44%) physicians did not specify a preference, and 5 (2.47%) physicians stated that they were neutral regarding evidence-based prescription guidelines for ABs.

### 3.11. Sources Consulted to Obtain Current/New Information on AB Therapy

The physicians reported using a range of resources to obtain up-to-date or new information about AB therapy, including the internet (generic websites), which 68 (33.66%) physicians preferred using. A low number of physicians (*n* = 13, 6.43%) relied on institutional recommendations, while the majority of physicians (*n* = 124, 61.38%) found such knowledge in textbooks. Moreover, 121 (59.9%) physicians reported utilizing the national clinical practice guidelines.

### 3.12. Additional Information Sources Considered Helpful for Reducing AB Resistance

In terms of additional information sources, 142 (70.29%) physicians suggested better clinical practice guidelines; 21 (10.39%) physicians recommended interactive case studies, training sessions, and websites with up-to-date information, news, and links; while 23 (11.38%) physicians stated that better access to current guidelines would be particularly helpful in relation to AB therapy.

### 3.13. Effects of Different Variables on Physicians’ Responses: A Regression Analysis

A multinomial logistic regression was performed to determine the effects of the physicians’ gender, age, area of specialization, sector of employment, visits to the clinic per quarter, and years of practice on the responses related to their daily work with AB resistance, AB prescribing behavior, instances of AB resistance in the region, strategy for delayed AB prescribing, discussion of AB resistance with infected patients, and evidence-based therapy guidelines for AB prescribing (Supplementary Material Supp S1).

According to the results of the multivariate regression analysis, the male gender substantially predicted statements that AB prescribing behavior had an effect on the development of AB resistance in the Hail region (OR: 0.35 [0.19–0.63], *p* ≤ 0.05) when compared with the female gender. In terms of the other responses, however, there was no difference.

The results also demonstrated that the age groups of 31–40 years (OR: 5.63 [1.12–43.52], *p* ≤ 0.05) and >50 years (OR: 2.31 [0.49–21.18], *p* ≤ 0.05) were significant in predicting the likelihood of physicians having encountered cases of AB resistance in the region.

Furthermore, the study results indicated that the specializations of obstetrics and gynecology (OR: 1.88 [0.73–4.23], *p* ≤ 0.05) and pediatrics (OR: 2.34 [1.01–12.52], *p* ≤ 0.05) significantly predicted statements that AB prescribing behavior affects the development of AB resistance in the Hail region. The specializations of cardiology (OR: 0.02 [0.03–0.14], *p* ≤ 0.05) and psychiatry (OR: 0.03 [0.05–0.41], *p* ≤ 0.05) were found to significantly predict responses related to the extent to which the physicians found their daily work to be associated with AB resistance. Additionally, the specializations of cardiology (OR: 0.22 [0.15–0.64], *p* ≤ 0.05) and psychiatry (OR: 0.07 [0.04–0.59], *p* ≤ 0.05) were also determined to significantly predict responses related to the physicians’ preference for more evidence-based therapy guidelines for AB prescribing.

Physicians making 301–500 visits to the clinic per quarter (OR: 3.23 [1.06–22.43], *p* ≤ 0.05) and those making 501–800 visits to the clinic per quarter (OR: 5.53 [0.52–54.43], *p* ≤ 0.05) significantly predicted the extent to which their daily work was associated with AB resistance. Moreover, physicians making 301–500 visits to the clinic per quarter (OR: 1.28 [0.94–5.34], *p* ≤ 0.05), 501–800 visits to the clinic per quarter (OR: 2.11 [1.01–11.40], *p* ≤ 0.05), and >1600 visits to the clinic per quarter (OR: 3.34 [1.16–19.93]) substantially predicted that AB prescribing behavior had an effect on the development of AB resistance in the Hail region.

In addition, physicians who have been in practice for 1–3 years (OR: 9.29 [1.08–47.06], *p* ≤ 0.05), 4–6 years (OR: 19.33 [1.03–57.29], *p* ≤ 0.05), 7–10 years (OR: 15.62 [2.25–36.22], *p* ≤ 0.01), and >10 years (OR: 12.85 [3.49–29.92], *p* ≤ 0.01) significantly predicted that AB prescribing behavior had an impact on the development of AB resistance in the Hail region. By contrast, no meaningful predictions for the other responses have been made.

## 4. Discussion

The effectiveness of ABs, which have saved millions of lives, is in jeopardy due to the rapid rise in AB resistance worldwide. Unfortunately, almost all ABs have eventually been met with resistance [21]. It has been proposed that a better understanding of what clinicians know and believe with regard to AB usage and resistance can help to improve the effectiveness of in-hospital AB use while minimizing AB resistance [22]. The questionnaire approach is considered an excellent tool for gathering data on physicians’ views and understanding of AB prescribing and resistance, which represent two of the key factors underpinning this global concern.

In order to learn about AB usage trends, the influence of physicians’ knowledge and attitudes toward ABs on the development of AB resistance, and physicians’ communication with patients regarding AB resistance in the Hail region of Saudi Arabia, we conducted a questionnaire-based survey among a variety of physicians, including GPs and specialists in pediatrics, psychiatry, cardiology, ophthalmology, and obstetrics and gynecology. AB therapy in primary and secondary care ought to make sense. We discovered that the attitudes of physicians in the Hail region towards prescribing ABs had a favorable effect. The Hail region’s population hardly ever experiences AB resistance, although ABs are nevertheless administered based on patients’ needs. When administering an AB, several physicians frequently brought up the issue of AB resistance with infected patients. However, a small number of physicians never inquired or expressed concern about AB resistance in patients with infections.

In our study, 101 physicians failed to discuss their opinions of AB resistance with patients to see if they were aware of the issue. Due to a lack of time and factors related to increasing patients’ concerns, 32 physicians did not discuss AB resistance with infected patients. It has been reported in earlier studies that physicians overprescribe ABs, which ultimately leads to AB resistance [23]. Physicians’ decision-making process concerning AB prescribing might differ according to patients’ and physicians’ perspectives on medical, cultural, and social practices [23]. Physicians must strictly follow the recommended guidelines for AB prescribing to prevent further complications. According to our findings, more than half of the physicians felt that patient pressure or parental expectations had no effect on their AB prescribing patterns. By contrast, it has been found that patient pressure is the most common factor influencing the AB prescribing pattern, even when physicians are aware that ABs are not appropriate for the relevant medical condition [23,24,25].

This study’s findings also revealed that 148 physicians believed AB resistance could be prevented by focusing on patients’ attitudes and knowledge of ABs. Even if physicians do not perceive AB resistance to be a significant problem in their practice, it should be considered when choosing ABs. Our results are consistent with those of research conducted in other contexts. For example, participants have reported that AB resistance is a significant issue, albeit less so in their individual practice than at the national and global levels [26,27]. Furthermore, 98 physicians in this study responded that they frequently employed a delayed AB prescribing method. AB resistance is a global problem. Importantly, hospitals lack treatment guidelines for managing infectious diseases in many countries and rely on international guidelines [28]. The lack of local hospital guidelines for managing patients with infectious diseases could prove lethal due to promoting irrational AB use, which could lead to further complications concerning AB resistance [28].

Whether due to delayed cultural findings or a lack of appropriate diagnostic techniques, ABs are frequently recommended based solely on personal experience. In our study, the majority of physicians often followed AB treatment practice guidelines in their day-to-day work, while a significantly lower proportion of physicians turned to textbooks and institutional guidelines for up-to-date or fresh material on AB therapy. In addition, some physicians (*n* = 68) preferred to find fresh material regarding AB therapy online via the internet, whereas 78 physicians preferred scholarly papers. The questionnaire used in this study should also be used to examine the factors that influence physicians’ prescribing behaviors in both hospital and primary care settings as well as to assess the relationships between the prescription of AB and other factors, such as sociodemographic or professional practice characteristics (e.g., medical specialization, workplace, workflow).

Our investigation has revealed the significant impact of physicians’ attitudes on AB prescribing in Saudi Arabia, which may be one of the reasons behind the increasing prevalence of infectious diseases and AB resistance. Our findings also indicate that hospitals in the Hail region have no standard AB prescribing procedures in place. This is a significant concern, as ABs should be prescribed properly in order to help with infection management and prevent the development of AB-resistant strains. AB prescribing committees should be implemented in hospitals where a culture of physician autonomy exists. Moreover, antimicrobial restrictions, via either the requirement for pre-authorization or formulary limitation and justification, should be implemented to effectively control AB use [29]. Education is very important in relation to the judicious use of ABs [30,31,32,33,34]. The most common active influence on hospital AB stewardship techniques is healthcare provider education. It is also critical to include antimicrobials in continuing medical education. Reducing inappropriate AB prescribing remains a critical goal in the fight against antimicrobial resistance.

## 5. Conclusions

This study demonstrated that physicians in the Hail region exhibited a comprehensive awareness of the elements that contribute to AB resistance, although they rarely communicated it to their patients, presuming the latter to be oblivious to the science behind AB resistance. The present findings suggest that the features influencing physicians’ AB prescribing behavior could be a powerful strategy for lowering AB resistance. The introduction of educational programs focused on AB resistance, with an emphasis on young physicians, might be a useful potential approach. Moreover, prudent antimicrobial usage should be promoted through educational and antimicrobial stewardship initiatives.

## Figures and Tables

**Table 1:** Validity and reliability test results.

Step A. Content and Validity		Step B. Reliability Analysis	
Content Search	Content Validity	Pilot Study	
Primary Stage - Literature review - Questionnaire development - Previous research published	Expert Review - Arabic translation of the questionnaire - Reviewed by a physician panel	Cronbach’s Analysis Physicians (*n* = 10) Alpha value = 0.73	Retest Analysis Physicians (*n* = 10) ICC value = 0.5

**Table 2:** Participant characteristics based on different age groups.

Parameters	Total 221 (100%)	Age Groups				*p*-Value
		25–30 Years 19 (9.5%)	31–40 Years 79 (39.3%)	41–50 Years 66 (32.8%)	>50 Years 37 (18.4%)	
**Gender**						
Male	124 (61.7)	10 (5.0)	48 (23.9)	38 (18.9)	28 (13.9)	0.238
Female	77 (38.3)	9 (4.5)	31 (15.4)	28 (13.9)	9 (4.5)	
**Specialization**						
General practitioner	70 (34.8)	18 (9.0)	34 (16.9)	11 (5.5)	7 (3.5)	<0.05
Internal Medicine	19 (9.5)	0	13 (6.5)	4 (2.0)	2 (1.0)	
Obstetrics and Gynaecology	24 (11.9)	0	6 (3.0)	8 (4.0)	10 (5.0)	
ENT	10 (5.0)	0	3 (1.5)	6 (3.0)	1 (0.5)	
Pulmonology	12 (6.0)	0	3 (1.5)	4 (2.0)	5 (2.5)	
Pediatrics	14 (7.0)	1 (2.0)	1 (2.0)	7 (3.5)	5 (2.5)	
Urology and Nephrology	12 (6.0)	0	6 (3.0)	5 (2.5)	1 (0.5)	
Cardiology	9 (4.5)	0	3 (1.5)	5 (2.5)	1 (0.5)	
Psychiatry	5 (2.5)	0	3 (1.5)	2 (1.0)	0	
Surgeon and General Surgery	3 (1.5)	0	1 (0.5)	0	2 (1.0)	
Plastic Surgery	4 (2.0)	0	2 (1.0)	1 (0.5)	1 (0.5)	
Anesthesiology	5 (2.5)	0	3 (1.5)	2 (1.0)	0	
Dermatology	3 (1.5)	0	0	1 (0.5)	2 (1.0)	
Ophthalmology	5 (2.5)	0	1 (0.5)	4 (2.0)	0	
Orthopedics	6 (3.0)	0	0	6 (3.0)	0	
**Sector of Employment**						
General—Specialized Hospitals	112 (56.3)	15 (7.5)	44 (22.1)	31 (15.6)	22 (11.1)	0.187
Govt Primary Care Centers	32 (16.1)	2 (1.0)	13 (6.5)	13 (6.5)	4 (2.0)	
Private Hospitals/Clinics	55 (27.6)	1 (0.5)	21 (10.6)	21 (10.6)	12 (6.0)	

**Table 3:** Physician visits per quarter (three months) to the clinic.

Visits per Quarter (Three Months) to the Clinic	Frequency	Percent	95% CI
≤100	27	13.36	2.25–16.3
101–300	46	23.10	5.12–24.7
301–500	50	25.10	5.78–31.3
501–800	38	19.10	0.84–19.7
801–1200	17	8.50	3.80–12.3
1201–1600	9	4.50	0.137–4.64
>1600	12	6.00	2.34–7.84

## Data Availability

Data is contained within the article or supplementary material.

## Supplementary Materials

The following supporting information can be downloaded at: https://www.mdpi.com/article/10.3390/healthcare11111576/s1: Supp S1: Specialization; Supp S2: Sector of employment; Supp S3: Frequency distribution of sector of employment; Supp S4: Frequency distribution of physician visits to the clinic per quarter; Supp S5: Daily work is associated with antibiotic resistance; Supp S6: Frequency of physicians whose daily work is associated with the AMR; Supp S7: Views on the impact of antibiotic prescribing behavior on the development of antibiotic resistance within the region; Supp S8: Frequency of physician’s antibiotic prescribing behaviors; Supp S9: Physician experience of AB resistance cases in the region; Supp S10: Frequency of antibiotics prescription; Supp S11: Discussion of the subject of antibiotic resistance with the patients suffering from infections; Supp S12: Frequency of discussion on AMR with patients; Supp S13: Usage of delayed antibiotic prescribing strategy; Supp S14: Usage of delayed antibiotic prescribing strategy; Supp S15: Practicing guidelines for antibiotic therapy during the daily work; Supp S16: Vote on having more evidence-based therapy guidelines for an antibiotic prescription; Supp S17: Working hours as a Physician (in years); Supp S18: Effect of different variables on the physician’s responses using regression analysis; Supp S19: Multinomial Logistic Regression.
