# Supplementary material for: Physician’s Knowledge and Attitudes on Antibiotic Prescribing and Resistance: A Cross-Sectional Study from Hail Region of Saudi Arabia
Source: Healthcare (Basel). 2023 May 27;11(11):1576. doi: 10.3390/healthcare11111576 (PMC10252466; doi:10.3390/healthcare11111576)
Supplement: Supplementary file 1 [file healthcare-11-01576-s001.zip › healthcare-2316263-supplementary.pdf]

Supplement file

Supp S1. Specialization

Specialization	n	Percent	95% CI
General practitioner (GP)	70	34.8	1.1-36
Internal Medicine	19	9.5	4.4-14
Obstetrics and Gynaecology	24	11.9	0.87-13
ENT	10	5.0	1.3-6.3
Pulmonology	12	6.0	0.44-6.4
Paediatrics	14	7.0	1.3-8.3
Urology	12	6.0	2.3-7.8
Cardiology	9	4.5	0.91-4.9
Psychiatry	5	2.5	1.8-4.3

Supp S2. Sector of employment

Sector of employment	Frequency (n)	Percent	95% CI
General or specialized governmental hospitals	112	56.3	6.63-82.6
Governmental primary care centre	32	16.1	1.36-26.3
Private Hospitals/Clinics	55	27.6	4.17-34.9

Supp S3. Frequency distribution of sector of employment

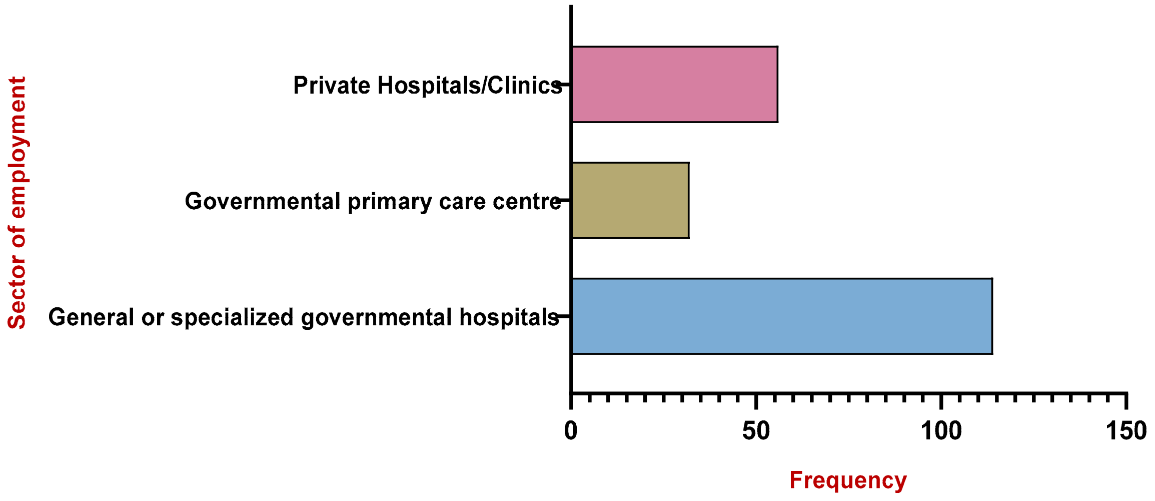

Supp S4. Frequency distribution of physician visits to the clinic per quarter

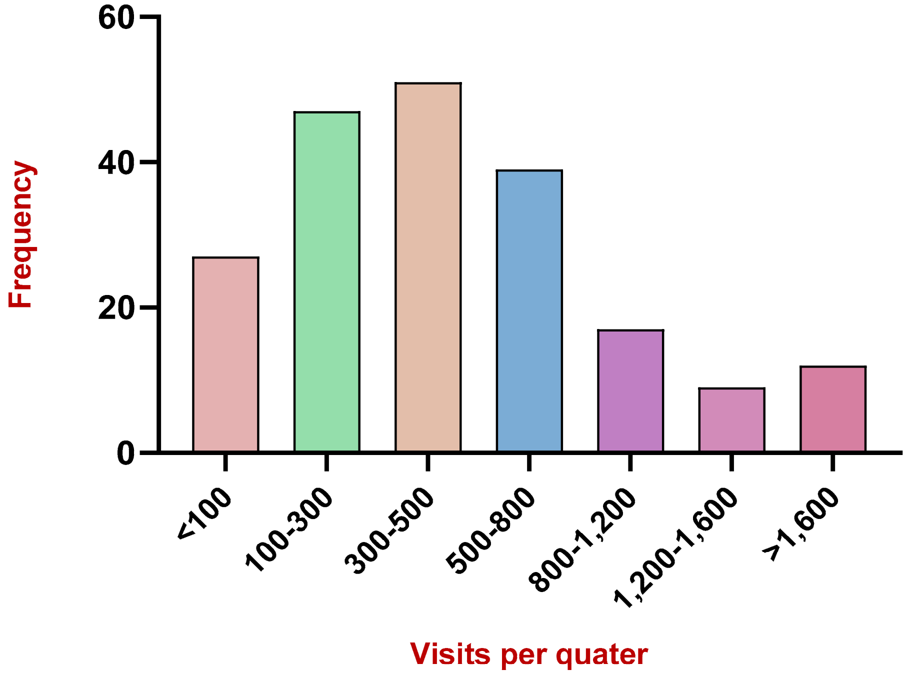

Supp S5. Daily work is associated with antibiotic resistance

Daily work is associated with antibiotic resistance	Frequency	Percent	95% CI
Slightly	78	38.61	10.3-66.92
Highly	25	12.37	0.911-13.4
Moderately	84	41.58	1.28-43.3
Not at all	15	7.42	1.36-13.48

Supp S6. Frequency of physicians whose daily work is associated with the AMR.

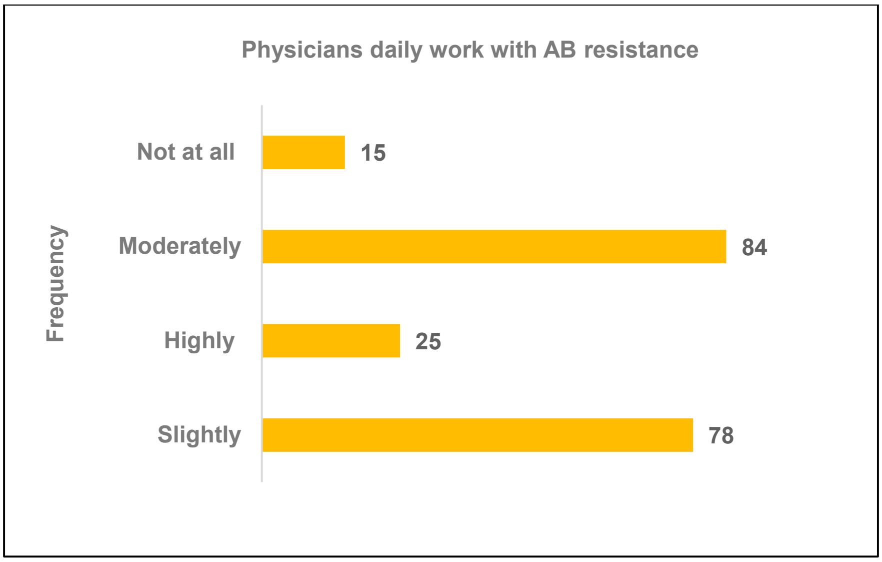

Supp S7. Views on the impact of antibiotic prescribing behavior on the development of antibiotic resistance within the region

Response	Frequency	Percent	95% CI
Yes	88	43.56	6.37-55.6
No	68	33.66	0.210-33.8
Don't know	46	22.77	6.55-38.99

Supp S8. Frequency of physician's antibiotic prescribing behaviors

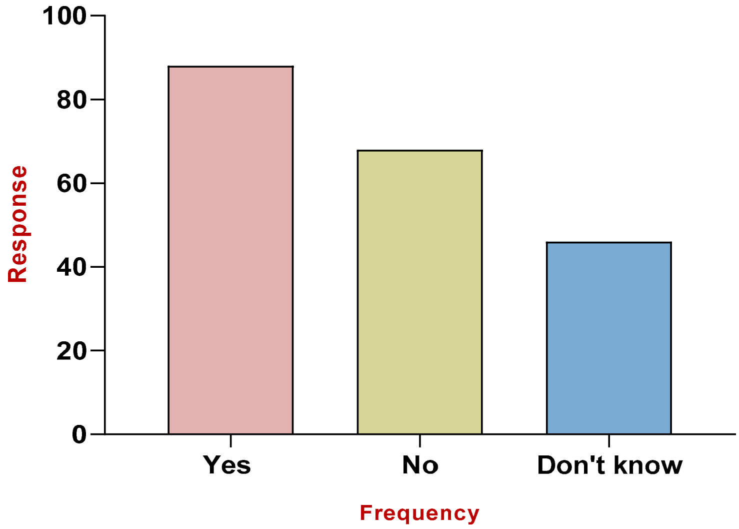

Supp S9. Physician experience of AB resistance cases in the region

Response	Frequency	Percent	95% CI
Never	19	9.40	1.01-13.1
Monthly	51	25.24	0.00-29.5
Rarely	104	51.48	9.41-58.7
Weekly	27	13.3	0.877-23.0
Daily	1	0.49	0.00-1.05

Supp S10. Frequency of antibiotics prescription

Frequency of antibiotic prescription	Frequency	Percent	95% CI
Daily	99	49	5.97-67.7
Weekly	73	36.13	6.61-41.2
Rarely	18	8.91	2.1-10.27
Monthly	12	5.94	1.17-11.8

Supp S11. Discussion of the subject of antibiotic resistance with the patients suffering from infections

Response	Frequency	Percent	95% CI
Rarely	31	15.34	3.39-27.29
Sometimes	85	42	4.39-67.2
Often	73	36.13	5.81-45.7
Never	13	6.4	1.41-11.39

Supp S12. Frequency of discussion on AMR with patients.

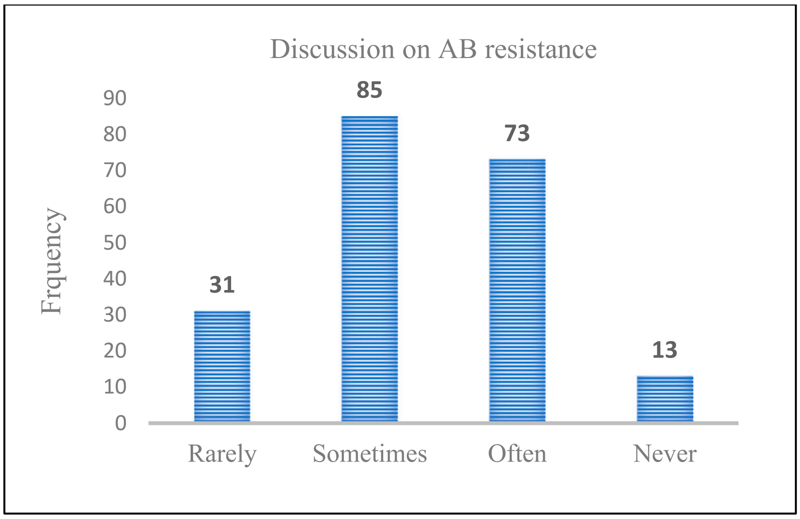

Supp S13. Usage of delayed antibiotic prescribing strategy

Response	Frequency	Percent	95% CI
Never	6	2.9	-1.55-4.55
Sometimes	98	48.5	9.59-89.41
Often	62	30.6	-4.48-35.5
Strategy not known	15	7.4	2.23-13.57
Rarely	21	10.39	3.25-19.53

Supp S14. Usage of delayed antibiotic prescribing strategy

Response	Frequency	Percent	95% CI
Never	6	2.9	-1.55-4.55
Sometimes	98	48.5	9.59-89.41
Often	62	30.6	-4.48-35.5
Strategy not known	15	7.4	2.23-13.57
Rarely	21	10.39	3.25-19.53

Supp S15. Practicing guidelines for antibiotic therapy during the daily work

Response	Frequency	Percent	95% CI
Frequently	101	50	0.17-50.3
Moderately	80	39.6	-4.82-44.8
Rarely	10	4.95	-2.09-7.09
No clear guidelines	11	5.4	-1.82-7.32

Supp S16. Vote on having more evidence-based therapy guidelines for an antibiotic prescription

Response	Frequency	Percent	95% CI
Yes	186	92.07	25.9-98.247
No	11	5.4	-0.5-6.03
Don't Know	05	2.47	-0.27-2.77

Supp S17. Working hours as a Physician (in years)

Working timeline	Frequency	Percent	95%CI
>10	110	54.45	6.91-61.9
7-10	43	21.28	12.2-33.7
4-6	32	15.84	8.38-24.4
1-3	14	6.93	4.56-11.6
<1	3	1.48	0.77-2.27

Supp S18. Effect of different variables on the physician's responses using regression analysis

Responses

Q7: To what extent do you find your daily work is associated with antibiotic resistance?

Q9: Do you think your antibiotic prescribing behavior has an impact on the development of antibiotic resistance within your region?

Q10: How frequently did you encounter instances of antibiotic resistance in the region?

Q12: How often do you use the strategy of delayed antibiotic prescribing? (not prescribing antibiotics immediately)

Q14: How frequently did you talk to patients who were infected about the issue of antibiotic resistance?

Q15: When do you discuss the subject of antibiotic resistance with your patients suffering from infections?

Q18: Would you like to have more evidence-based therapy guidelines for antibiotic prescription?

For all the tables

*≤0.05

**≤0.01

Supp S19. Multinomial Logistic Regression

Variables	Total (N)	Q7	Q9	Q10	Q12	Q14	Q15	Q18
**Gender**								
Female	77(38.3)	Ref	Ref	Ref	Ref	Ref	Ref	Ref
Male	124(61.7)	1.06 (0.36 – 3.11)	0.35 (0.19 – 0.63)*	1.48 (0.57 – 3.84)	0.31 (0.03 – 2.69)	2.72 (0.85 – 8.63)	1.66 (0.63 – 4.40)	0.95 (0.33 – 2.72)
**Age**								
25 – 30 years	19(9.5)	Ref	Ref	Ref	Ref	Ref	Ref	Ref
31 – 40 years	79(39.3)	1.07 (0.23 – 2.49)	1.15 (0.41 – 3.16)	10.26 (1.72 – 61.2)*	3.78 (2.13 – 6.24)	2.98 (0.46 – 19.23)	1.04 (0.20 – 5.36)	2.28 (0.51 – 10.11)
41 – 50 years	66(32.8)	0.94 (0.19 – 2.11)	1.07 (0.38 – 3.02)	1.33 (0.37 – 4.79)	2.56 (2.04 – 5.22)	1.43 (0.25 – 8.06)	3.76 (0.49 – 28.71)	2.28 (0.49 – 10.60)
More than 50 Years	37(18.4)	0.38 (0.09 – 1.72)	0.89 (0.29 – 2.74)	4.80 (0.79 – 29.07)*	1.18 (1.89 – 2.73)	1.37 (0.20 – 9.001)	0.62 (0.11 – 3.45)	3.37 (0.51 – 22.19)
**Area of Specialization**								
General practitioner (GP)	70(34.8)	Ref	Ref	Ref	Ref	Ref	Ref	Ref
Internal Medicine	19(9.5)	7.34 (8.34 – 5.12)	1.44 (0.52 – 4.01)	4.22 (1.98 – 5.23)	3.12 (2.02 – 5.28)	1.07 (0.23 – 2.49)	1.18 (0.28 – 4.87)	2.43 (0.28 – 3.28)
Obstetrics and Gynecology	24(11.9)	1.04 (0.10 – 10.55)	2.61 (0.98 – 6.88)*	2.81 (1.75 – 4.33)	1.27 (2.19 – 3.12)	1.48 (0.57 – 3.84)	1.33 (0.37 – 4.79)	0.85 (0.15 – 4.75)
ENT	10(5.0)	0.18 (0.02 – 1.25)	1.31 (0.34 – 4.91)	1.22 (1.45 – 3.23)	2.18 (4.12 – 3.23)	3.78 (2.13 – 6.24)	1.04 (0.20 – 5.36)	3.28 (1.27 – 4.58)
Pulmonology	12(6.0)	0.50 (0.04 – 5.25)	0.92 (0.26 – 3.21)	1.87 (1.27 – 2.11)	1.38 (2.19 – 3.57)	1.93 (0.68 – 5.42)	1.70 (0.29 – 9.97)	2.19 (0.37 – 3.92)
Pediatrics	14(7.0)	7.14 (8.31 – 5.52)	4.76 (1.22 – 18.61)*	1.94 (2.34 – 3.26)	1.13 (1.19 – 2.83)	0.38 (0.09 – 1.72)	0.95 (0.33 – 2.72)	0.46 (0.81 – 2.70)
Urology	12(6.0)	7.22 (8.18 – 5.36)	3.58 (1.68 – 4.87)	2.23 (1.78 – 4.29)	2.03 (1.23 – 3.76)	1.06 (0.36 – 3.11)	2.28 (0.51 – 10.11)	0.27 (1.28 – 2.10)
Cardiology	9(4.5)	0.02 (0.04 – 0.17)*	0.18 (0.02 – 1.59)	0.67 (1.35 – 2.18)	1.47 (2.86 – 3.27)	2.56 (2.04 – 5.22)	1.48 (0.57 – 3.84)	0.13 (0.24 – 0.71)*
Psychiatry	5(2.5)	0.06 (0.08 – 0.57)*	0.86 (0.13 – 5.52)	0.36 (1.32 – 2.27)	0.26 (1.27 – 2.18)	1.37 (0.20 – 9.001)	1.07 (0.38 – 3.02)	0.11 (0.01 – 0.87)*
Others	30(14.8)	1.31 (0.13 – 13.21)	0.47 (0.18 – 1.21)	0.93 (1.42 – 2.31)	0.75 (1.11 – 2.85)	0.89 (0.29 – 2.74)	0.35 (0.19 – 0.63)	1.09 (0.21 – 5.98)
**Sector of Employment**								

General or specialized governmental hospitals	112(56.3)	Ref	Ref	Ref	Ref	Ref	Ref	Ref
Governmental primary care center	32(16.1)	2.98 (0.36 – 24.21)	1.47 (0.67 – 3.24)	1.13 (0.22 – 5.61)	1.03 (0.17 – 3.26)	0.35 (0.07 – 1.65)	0.46 (0.14 – 1.49)	0.92 (0.24 – 3.60)
Private Hospitals/Clinics	55(27.6)	1.25 (0.37 – 4.17)	1.28 (0.67 – 2.44)	0.39 (0.14 – 1.08)	0.23 (0.04 – 1.30)	0.30 (0.08 – 1.12)	1.11 (0.32 – 3.78)	1.69 (0.44 – 6.43)
**Visits per quarter in the clinic**								
<100	27(13.3)	Ref	Ref	Ref	Ref	Ref	Ref	Ref
100-300	46(23.1)	2.44 (0.59 – 10.02)	1.93 (0.68 – 5.42)	1.87 (0.42 – 8.17)	0.92 (0.26 – 3.21)	1.76 (2.48 – 4.32)	1.46 (3.26 – 3.47)	2.19 (1.28 – 3.58)
300-500	50(25.1)	5.56 (1.02 – 30.94)*	2.74 (0.99 – 7.62)*	2.78 (0.57 – 13.47)	1.43 (2.58 – 3.67)	2.45 (3.57 – 1.27)	2.67 (0.65 – 10.92)	0.23 (2.34 – 3.47)
500-800	38(19.1)	8.63 (0.94 – 78.75)*	1.78 (0.60 – 5.23)	1.18 (0.28 – 4.87)	1.94 (2.34 – 3.26)	3.22 (5.18 – 3.36)	1.25 (0.33 – 4.60)	0.47 (2.19 – 3.28)
800-1,200	17(8.5)	1.70 (0.29 – 9.97)	4.08 (1.11 – 14.88)*	2.78 (0.28 – 27.26)	2.96 (3.29 – 1.86)	1.22 (1.45 – 3.23)	1.06 (0.21 – 5.15)	0.87 (1.27 – 2.38)
1,200-1,600	9(4.5)	1.81 (0.18 – 18.03)	2.28 (0.47 – 11.01)	3.14 (0.42 – 33.42)	1.31 (0.34 – 4.91)	1.43 (0.25 – 8.06)	1.18 (1.89 – 2.73)	0.93 (1.28 – 2.19)
>1,600	12(6.0)	1.44 (0.12 – 14.16)	5.71 (1.30 – 25.02)*	0.87 (0.13 – 5.54)	0.92 (0.26 – 3.21)	0.18 (0.02 – 1.25)	0.62 (0.11 – 3.45)	0.74 (1.96 – 2.38)
**Years of practice**								
<1	3(1.5)	Ref	Ref	Ref	Ref	Ref	Ref	Ref
1 – 3	14(6.9)	8.21 (2.34 – 12.85)	13.71 (1.16 – 61.34)*	3.21 (2.47 – 6.34)	1.15 (0.41 – 3.16)	0.31 (0.03 – 2.69)	1.38 (2.19 – 3.57)	5.61 (0.46 – 61.04)
4 – 6	32(15.8)	12.89 (3.77 – 21.32)	26 (1.11 – 64.49)*	12.73 (2.23 – 17. 12)	0.95 (0.33 – 2.72)	4.80 (0.79 – 29.07)	1.48 (0.57 – 3.84)	1.25 (0.08 – 17.97)
7 – 10	43(21.3)	9.22 (2.56 – 11.92)	22.34 (2.74 – 41.53)**	3.83 (2.47 – 12. 11)	1.22 (1.45 – 3.23)	0.62 (0.11 – 3.45)	1.31 (0.13 – 13.21)	1.37 (0.14 – 21.44)
>10	110(54.4)	10.18 (3.84 – 13.49)	18.44 (3.73 – 39.89)**	4.91 (3.26 – 7.83)	3.28 (1.27 – 4.58)	2.81 (1.75 – 4.33)	1.43 (0.25 – 8.06)	10.25 (0.63 – 16.65)
